# Localized Structural Alterations Underlying a Subset of Unexplained Sudden Cardiac Death

**DOI:** 10.1161/CIRCEP.117.006120

**Published:** 2018-07-12

**Authors:** Michel Haïssaguerre, Mélèze Hocini, Ghassen Cheniti, Josselin Duchateau, Frédéric Sacher, Stéphane Puyo, Hubert Cochet, Masateru Takigawa, Arnaud Denis, Ruairidh Martin, Nicolas Derval, Pierre Bordachar, Philippe Ritter, Sylvain Ploux, Thomas Pambrun, Nicolas Klotz, Gregoire Massoullié, Xavier Pillois, Corentin Dallet, Jean-Jacques Schott, Solena Scouarnec, Michael J. Ackerman, David Tester, Olivier Piot, Jean-Luc Pasquié, Christophe Leclerc, Jean-Sylvain Hermida, Estelle Gandjbakhch, Philippe Maury, Louis Labrousse, Ruben Coronel, Pierre Jais, David Benoist, Edward Vigmond, Mark Potse, Richard Walton, Koonlawee Nademanee, Olivier Bernus, Remi Dubois

**Affiliations:** 1 IHU Liryc, Electrophysiology and Heart Modeling Institute, Fondation Bordeaux Université, France (M. Haïssaguerre, M. Hocini, G.C., J.D., F.S., S.P., H.C., M.T., A.D., R.M., N.D., P.B., P.R., S.P., T.P., N.K., G.M., X.P., C.D., L.L., R.C., P.J., D.B., E.V., M.P., R.W., O.B., R.D.).; 2 Univ. Bordeaux (M. Haïssaguerre, M. Hocini, J.D., F.S., S.P., H.C., A.D., N.D., P.B., P.R., S.P., P.J., D.B., R.W., O.B., R.D.); 3 INSERM, Centre de recherche Cardio-Thoracique de Bordeaux, France (M. Haïssaguerre, M. Hocini, J.D., F.S., S.P., H.C., A.D., N.D., P.B., P.R., S.P., P.J., D.B., R.W., O.B., R.D.).; 4 Univ. Bordeaux, IMB UMR 5251, CNRS (E.V.).; 5 CNRS, IMB, UMR5251, Talence (E.V.).; 6 Bordeaux University Hospital (CHU), Cardiac Electrophysiology and Cardiac Stimulation Team, Pessac, France (M. Haïssaguerre, M. Hocini, G.C., J.D., F.S., S.P., H.C., M.T., A.D., N.D., P.B., P.R., S.P., T.P., N.K., G.M., X.P., L.L., P.J.).; 7 Pacific Rim Electrophysiology Research Institute, White Memorial Medical Center, Los Angeles, CA (K.N.).; 8 Inserm UMR 915 l’institut du thorax IRT, Nantes Cedex, France (J.-J.S., S.L.S.).; 9 Sudden Death Genomics Laboratory, Mayo Clinic, Rochester, MN (M.J.A., D.T.).

**Keywords:** catheter ablation, endocardium, epicardial mapping, incidence, sudden cardiac death, ventricular fibrillation

## Abstract

**Background:**

Sudden cardiac death because of ventricular fibrillation (VF) is commonly unexplained in younger victims. Detailed electrophysiological mapping in such patients has not been reported.

**Methods:**

We evaluated 24 patients (29±13 years) who survived idiopathic VF. First, we used multielectrode body surface recordings to identify the drivers maintaining VF. Then, we analyzed electrograms in the driver regions using endocardial and epicardial catheter mapping during sinus rhythm. Established electrogram criteria were used to identify the presence of structural alterations.

**Results:**

VF occurred spontaneously in 3 patients and was induced in 16, whereas VF was noninducible in 5. VF mapping demonstrated reentrant and focal activities (87% versus 13%, respectively) in all. The activities were dominant in one ventricle in 9 patients, whereas they had biventricular distribution in others. During sinus rhythm areas of abnormal electrograms were identified in 15/24 patients (62.5%) revealing localized structural alterations: in the right ventricle in 11, the left ventricle in 1, and both in 3. They covered a limited surface (13±6 cm^2^) representing 5±3% of the total surface and were recorded predominantly on the epicardium. Seventy-six percent of these areas were colocated with VF drivers (*P*<0.001). In the 9 patients without structural alteration, we observed a high incidence of Purkinje triggers (7/9 versus 4/15, *P*=0.033). Catheter ablation resulted in arrhythmia-free outcome in 15/18 patients at 17±11 months follow-up.

**Conclusions:**

This study shows that localized structural alterations underlie a significant subset of previously unexplained sudden cardiac death. In the other subset, Purkinje electrical pathology seems as a dominant mechanism.

## Abstract 2

Supplemental Digital Content is available in the text.

**WHAT IS KNOWN?**

- Unexplained sudden cardiac death or idiopathic ventricular fibrillation (VF, when an arrhythmia has been documented) is defined as no apparent heart disease after extensive investigations. It remains the puzzling conclusion for 5% to 14% of sudden deaths, with a higher incidence in young adults (up to 40%).
- VF is driven by continuous formation of reentrant waves, of which a critical determinant is the presence of structural heterogeneities.

**WHAT THE STUDY ADDS?**

- VF drivers can be localized by body surface mapping in humans.
- Catheter-based endo- and epicardial mapping in survivors of idiopathic VF enabled detection of abnormal electrogram areas indicative of localized myocardial abnormality; the abnormal areas colocalize with VF drivers suggesting a mechanistic role.
- In patients without myocardial abnormality, Purkinje excitability is mostly present.

Sudden cardiac death (SCD) remains a daunting health problem in all continents with estimates varying between 180 000 and 350 000 victims per year in the United States or in Europe.^1–6^ Coronary artery disease and cardiomyopathies are the main causes in older persons. However, in victims younger than 35 years, a common finding is the absence of structural heart disease at autopsy, reported in 29% to 40% of cases in recent population-based studies.^7–12^ Among patients surviving after resuscitation maneuvers, ventricular fibrillation (VF) is consistently the lethal heart rhythm disorder identified at the time of event.^3,6,12^ Over the last 20 years, considerable efforts have been devoted to the search for discrete electrocardiographic or imaging signs and genetic markers in survivors. Studies involving a wide panel of cardiac ion-channel genes have resulted in the discovery of new inherited electrical syndromes. In addition, numerous cardiomyopathy-related variants of uncertain significance have been reported, but the pathogenicity of most variants (eg, their capability of producing an arrhythmogenic phenotype) has not been validated by functional studies.^3,13,14^

Currently, unexplained SCD or idiopathic VF (when an arrhythmia has been documented^15–17^) is defined as no apparent structural or electrical heart disease after extensive investigations (eg, no phenotype). It remains the final conclusion for 5% to 14% of SCD cases in the global population, with a higher incidence in young adults.^1–3,6,12^ Reasons that prevent further understanding of these puzzling deaths may include the inability of current imaging techniques to identify subtle structural alterations; the lack of mapping detail during these lethal arrhythmia in humans; and unknown acute or dynamic phenomena. In animal and theoretical models using structurally normal hearts, VF is difficult to provoke via either electrical or pharmacological means. When induced, fibrillation is maintained by focal or reentrant spiral waves originating from multiple areas with a widespread cardiac distribution.^18,19^ Here, we report results of VF mapping in humans surviving idiopathic VF. We located the drivers maintaining VF from a panoramic array of 252 body surface electrodes.^20–22^ Then, we analyzed the electrograms present at the driver locations on the endocardium and epicardium during sinus rhythm. The findings provide evidence that localized areas indicating subclinical structural abnormalities may underlie a significant part of previously unexplained SCD.

## Methods

The data, analytic methods, and study materials will not be made available to other researchers for purposes of reproducing the results or replicating the procedure.

### Clinical Cases

Between January 2013 and December 2017, 24 patients surviving unexplained sudden death were consecutively included (Table 1). All were victims of instantaneous cardiac arrest with a witness beginning resuscitation until rescues documented VF and performed defibrillation.

**Table 1.**
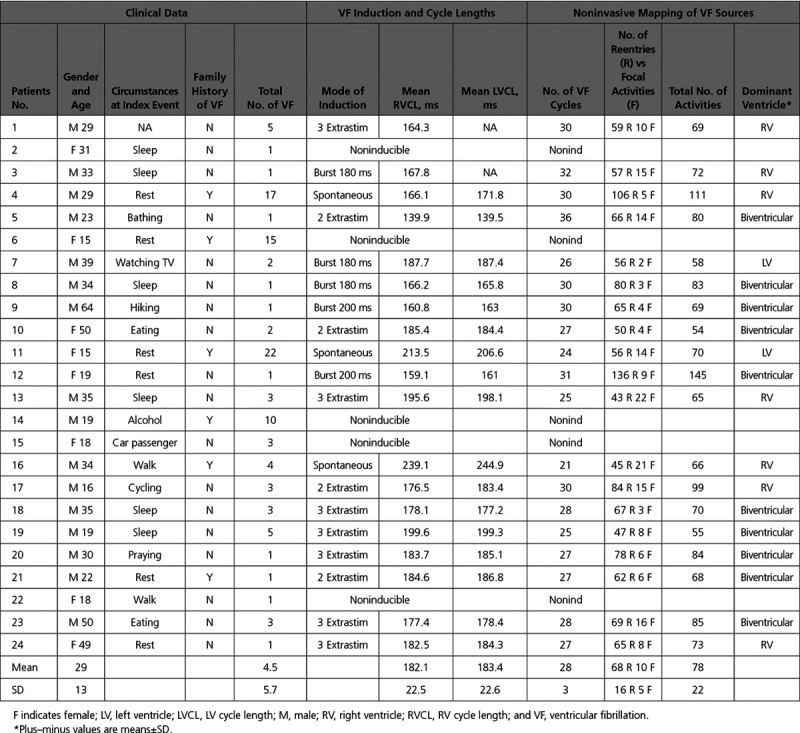
Sample Characteristics and Mapping Result

On the basis of published guidelines, patients were classified as having idiopathic VF as they had no clinical evidence for drug intoxication or electrolyte abnormality at the time of initial presentation, no identifiable structural heart disease demonstrated by normal echocardiographic and delayed gadolinium-enhanced magnetic resonance imaging, no detectable coronary artery disease on coronary angiography or exercise testing, and no known repolarization abnormalities associated with long or short QT interval or J wave. Pharmacological testing with an infusion of a sodium-channel blocker (ajmaline) or catecholamine (adrenaline and isoprenaline) was performed to exclude Brugada syndrome, long QT, and catecholaminergic polymorphic ventricular tachycardia, respectively.^6,23,24^

Genetic testing was performed in 17 patients. Sequencing was performed on a set of 98 arrhythmia and cardiomyopathy genes using the Haloplex capturing system in 13 patients, and other 4 were tested for long QT genes (including SCN5A) by standard Sanger sequencing. Among those genes, only 1 patient (number 15) had a likely pathogenic mutation in SCN5A and 1 in RyR2 (number 17). None of them had significant changes observed during exercise testing, isoproterenol testing, and ajmaline testing.

All patients had received an implantable defibrillator that provided accurate information on recurrence of VF. Fourteen patients were referred for recurrent arrhythmia during a postimplantation period of 36±12 months; these patients had failed at least one antiarrhythmic medication (including quinidine in 7, β-blocker in 6, and amiodarone in 3). Ten cases were referred after their index event. During hospitalization, 9 patients had ventricular ectopy (7 with short-coupling interval <320 ms) documented by 12-lead ECGs, whereas 15 patients had no ectopy; in 2 of the latter, ectopy appeared during pharmacological testing.

### Electrophysiological Study

The study was approved by the Institutional Clinical Research and Ethics Committee and all patients gave written informed consent. First, we identified the activities driving VF using body surface recordings. Second, intracardiac catheter mapping was performed during sinus rhythm to obtain high-density recordings of endocardial and epicardial electrograms. The flow diagram is shown in Figure I in the Data Supplement.

#### Noninvasive Mapping During VF

**Figure 1.**
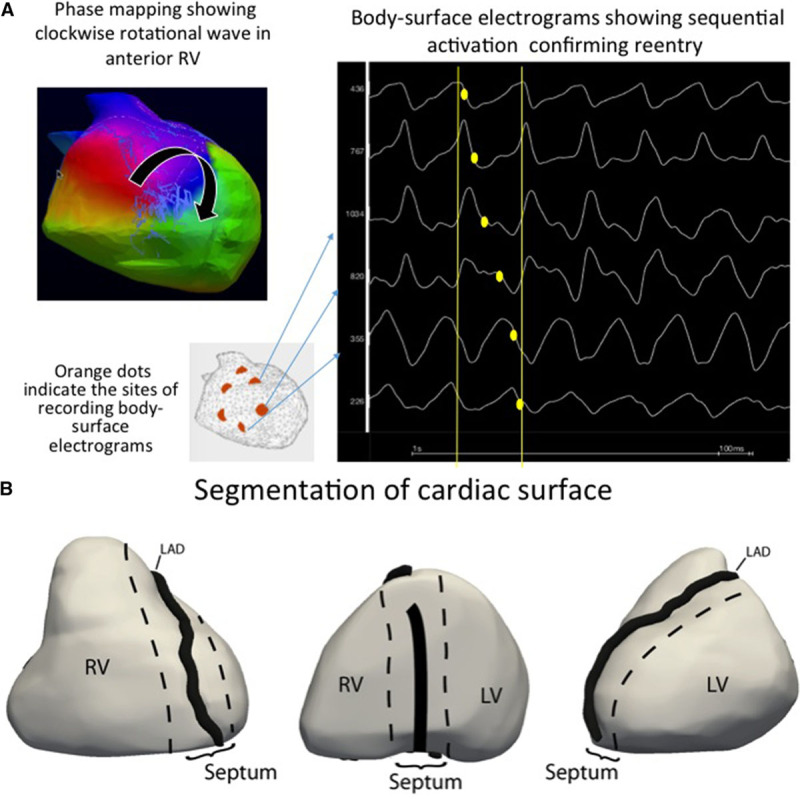
**Identification and location of reentrant drivers. A, Phase mapping and activation mapping. Left**, Shows the color-coded phases of wave propagation. Reentrant activity is shown as full phase progression around a center-point in the anterior right ventricle (RV). **Right**, Shows raw unipolar electrograms recorded around the reentry trajectory which indicate corroborated sequential activation. **B**, Regions of epicardial heart surface. The epicardial surface is divided into 3 regions to describe anatomic distribution of driver activity. 1, Right ventricle. 2, Septum (interventricular groove). 3, Left ventricle (LV). LAD indicates left anterior descending artery.

Body surface mapping was performed by reconstructing cardiac epicardial potentials from the chest using an array of 252 electrodes^20,21^ combined to computed tomography-based geometry (ECVue, Medtronic, OH), to provide a macroscopic view of ventricular wave fronts and identify the drivers at the origin of wave fronts. Unipolar electrograms were reconstructed from each node of an unstructured mesh obtained from computed tomographic images. Phase was calculated at each node using a dedicated Hilbert transform to identify activation wave fronts. Because of potential limitations of phase mapping in detection of reentry,^22,25^ we also analyzed the pattern of electrogram activation to confirm reentry by sequential activation of electrograms (Figure 1A). However, as with continuing fibrillation, electrograms began to split precluding their exact annotation, only the initial 5 seconds showing unaltered electrograms were analyzed. The mean VF cycle lengths during 5 seconds were measured on body surface from both ventricles.

The activities driving VF were either focal breakthrough, where activation started from a discrete point or reentry, where a wave front rotated on itself within a localized area. The mean number of activities per VF cycle was defined as the total number of activities divided by the number of VF cycles. The drivers were located on the ventricular surface distributed into 3 compartments (Figure 1B; Figure II in the Data Supplement): right ventricle, left ventricle, and septum (the surface of the interventricular groove). A compartment harboring >50% of a total number of driver activities was considered as dominant.

#### Intracardiac Mapping

Invasive electrophysiological testing was performed with the use of 3 multielectrode catheters introduced percutaneously through the femoral vessels. Surface electrocardiographic leads and intracardiac electrograms were recorded and stored on a recorder system.

Programmed ventricular stimulation was performed in 21 patients to assess inducibility of VF and exclude ventricular tachycardia, whereas the remaining 3 patients had spontaneous VF. Pacing was performed from right ventricular apex using up to 3 premature stimuli, and optionally from the left ventricle. The arrhythmia was recorded during the defibrillator charging period before cardioversion. Endocardial fibrillation cycle lengths were measured from the right ventricular catheter.

Electroanatomical mapping of the endocardium and epicardium was performed during sinus rhythm to identify healthy tissue from sites of structural alteration based on electrogram characteristics.^26–30^ A transseptal or retroaortic approach was performed to access the endocardial left ventricle and a subxyphosternal approach to access into the pericardial space. Decapolar catheters were used for endocardial mapping and multispline catheter (Pentaray, BiosenseWebster, CA) for epicardial measurements; all catheters had the same interelectrode distance of 2 mm, selected to minimize recording of far-field potentials. Electrogram criteria were identical to those defining fibrotic and cardiomyopathic tissue during mapping of ischemic or dilated cardiomyopathies.^26–30^ In the present study, we used the term structural alteration, as the specific affection responsible for electrogram fractionation (altering myocardial cells or their connections, fibrotic, or fatty tissue) was undetermined. Areas of low-amplitude electrograms were delineated in bipolar (<1 mV) and unipolar modes (<8.3 and 5.5 mV in left and right ventricles, respectively) on 3-dimensional ventricular reconstruction (CARTO system, Biosense Webster). However, because low-amplitude electrograms can be because of normal fat tissue or vasculature on the epicardium, electrograms were only considered abnormal if they harbored fragmented signals with >3 components and a duration superior to 70 ms. Greater mapping density was acquired in the cases of abnormality to delineate the abnormal surface area.

The spatial relationship between structurally abnormal areas and VF drivers was evaluated by comparing their mutual distances. The electroanatomical mapping was registered to the biventricular mesh obtained from the computed tomographic scan, which was composed of 1406±17 nodes and included the VF driver regions. The geodesic distances from nodes with, and from nodes without abnormal electrograms, to the closest driver region border were compared (Figure III in the Data Supplement).

#### Mapping of VF Triggers

The ECG recordings performed at the time of hospitalizations including after the initial cardiac arrest were obtained to look for morphological characteristics of ventricular premature beats, including their coupling interval, and their ability to trigger VF.^31^ Spontaneous ectopy (if present) were mapped to confirm Purkinje origin by the presence of sharp Purkinje potential preceding by <15 ms the ventricular electrogram during sinus rhythm and preceding ventricular activation during premature beats. Its absence at the site of earliest activation indicated an origin from ventricular muscle.

### Ablation

Spontaneous VF triggers (if present) were targeted as described previously.^31^ Substrate ablation was performed only in patients with recurrent VF episodes. An irrigated–tip ablation catheter was used with the target of eliminating fragmented electrograms.^26–28,30^ Radiofrequency energy was delivered with a power of 30 to 40 W and duration varying from 15 to 60 seconds guided by the impact on the local electrogram. The temperature was limited to 45°C. Radiofrequency lesions were delivered point-by-point at the area covering abnormal electrograms using serial applications. The patients were followed-up every 6 months by their individual physicians for clinical review and device interrogation.

### Statistical Methods

Continuous variables were reported as means±SD or medians (25th and 75th percentiles), as appropriate. Categorical variable (prevalence of Purkinje triggers) was compared with Fisher exact test. The geodesic distances of VF driver areas to structurally normal and abnormal areas were compared with a 2-sided Mann-Whitney test. Statistical significance was established at *P*≤0.05. All statistical analyses were performed using SPSS version 21.0 (SPSS Inc, Chicago, IL).

## Results

The main results are summarized in Tables 1 and 2. The patients were mostly male with VF occurring during sleep or rest, similar to others reports of unexplained SCD.^9^

### Driving Activities During VF

VF occurred spontaneously in 3 patients and was induced by electrical stimulation in 16, whereas it was noninducible in 5 patients.

**Figure 2.**
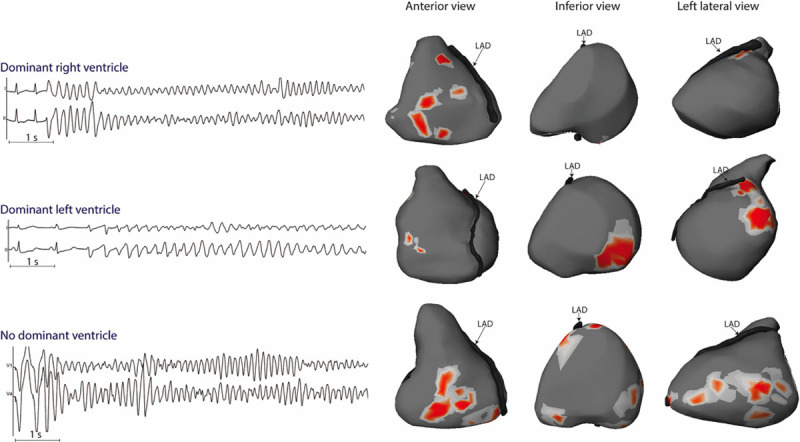
**Location of driver regions in 3 patients.** ECG examples of ventricular fibrillation (VF) onset (Stars) are shown from 3 patients (from **top** to **bottom**, patients no. 4, 11, and 5): the **upper** 2 patients (A) and (B) had spontaneously occurring VF in-hospital and case (C) had VF induced by electrical stimulation. Maps are shown on the body surface ventricular projection on anterior, inferior, and left lateral views. The locations of reentries are shown in red and vary among the 3 patients. Patient A presents drivers dominantly located in the right ventricle; patient B harbors drivers dominantly in the left ventricle (**middle**), whereas patient C shows a biventricular distribution (no dominant ventricle). Left descending artery (LAD) on the surface of the interventricular groove separating right and left ventricle.

A mean of 28±3 fibrillation cycles was analyzed in the initial 5 seconds of arrhythmia. The mean VF cycle length was 183±23 ms. VF was maintained by reentrant or focal activity in all patients. However, a limited number of activities coexisted with a mean of 2.8±0.7 per VF cycle. Reentry was the main activity driving VF compared with focal activity (87% versus 13%). It varied temporally from single occurrence to several repetitive rotations. In 9 of 19 patients, the driving activities were dominantly located in one ventricle, which harbored 59±9% of total activities (the right ventricle in 7 and the left ventricle in 2); whereas there were evenly distributed in other patients (Figure 2; Table 1). Two examples of VF activity are provided as Movies I and II in the Data Supplement. In 3 patients with a dominant ventricle, we mapped a second arrhythmia episode (spontaneous in 1, or induced by left-sided pacing in 2) that confirmed that VF was driven from the same ventricle.

### Abnormal Electrogram Areas During Sinus Rhythm

**Figure 3.**
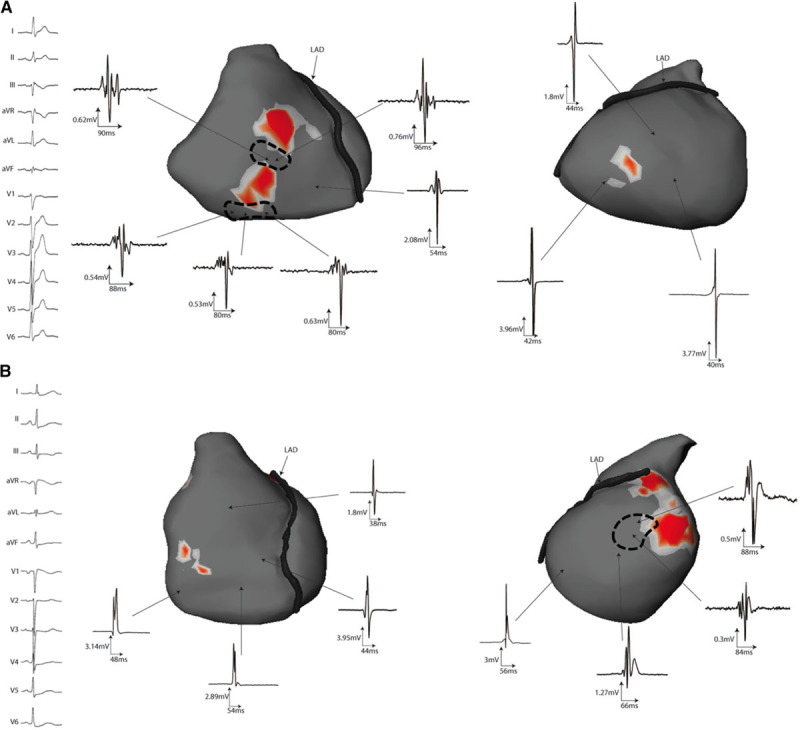
**Spatial location of abnormal signal areas with those of the drivers identified during ventricular fibrillation (VF).** Twelve-lead ECGs and intracardiac electrograms are recorded during sinus rhythm in 2 patients. The maps indicate the regions of reentry activities during VF and the clusters of abnormal electrograms in sinus rhythm are indicated by the dotted contour. Note the prolonged fragmented electrograms within an overall low-voltage electrogram. **A**, Patient 14, epicardial electrograms shows 2 abnormal areas over the anterior and inferior right ventricle, in close proximity with location of reentry activities, whereas other regions did not show abnormal electrograms. **B**, Patient 4, recordings from the right and left ventricles show colocation of abnormal electrograms in sinus rhythm, with the main VF driver in the posterior left ventricle. LAD indicates left descending artery.

A mean of 594±402 (endocardial right ventricle), 573±281 (endocardial left ventricle), and 2153±1242 (epicardium) sites were recorded per patient. Areas of abnormal electrograms were found in 15 of 24 pts (62.5%) patients. They were arranged in confluent (rather than sparse) distribution indicating localized structural alterations. The longest electrogram duration per patient was 89±10 ms. The abnormal areas were found in the right ventricle in 11 patients, the left ventricle in 1 and both in 3 (Table 2). They covered a limited surface area per patient of 13±6 cm2 (median, 12.5; interquartile range [IQR], 5–24 cm2) representing 5±3% of the total ventricular surface (median, 4; IQR, 3.2%–7.8%). They were surrounded by a border zone harboring fractionated signals not fulfilling pathological criteria (Figures 3 through 5; Figure IV in the Data Supplement). The comparison of endocardial and epicardial recordings at the same locations showed that the abnormal electrograms were largely confined in one ventricular side (endocardial in 2 patients, epicardial in 12, both in 1) indicating that the pathology involved a part of the ventricular wall rather than being transmural.

**Table 2.**
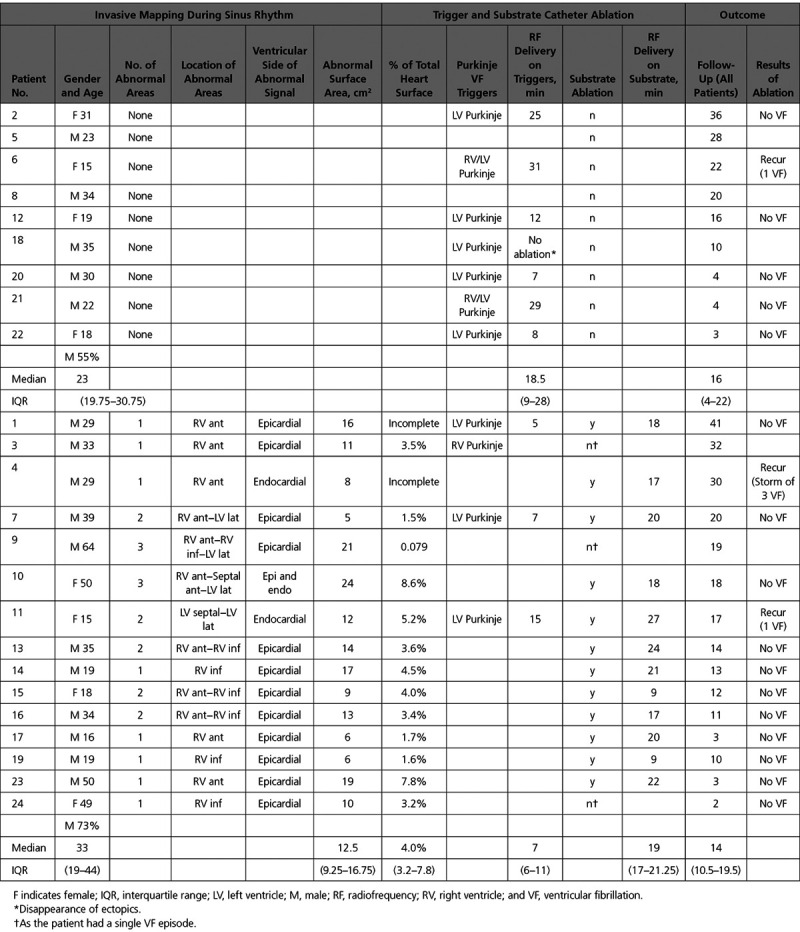
Invasive Mapping and Ablation

**Figure 4.**
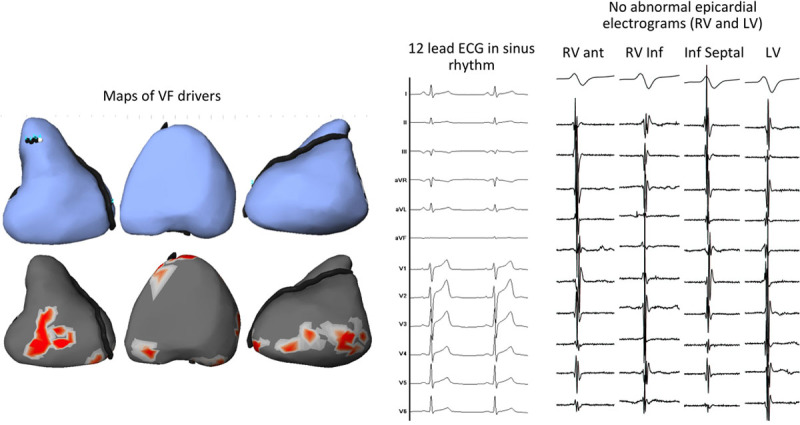
**Example of healthy signals during sinus rhythm (patient no. 5). Left**, Maps, in anterior, inferior, and left lateral views, show focal breakthroughs (blue points in **upper** blue) and reentry areas (red areas in **lower**). The pattern of ventricular fibrillation (VF) activities shows no specific distribution. **Middle**, Shows the 12-lead ECG in sinus rhythm. **Right**, Shows the presence of normal narrow signals in all sites of epicardium indicating healthy local tissue. LV indicates left ventricle; and RV, right ventricle.

**Figure 5.**
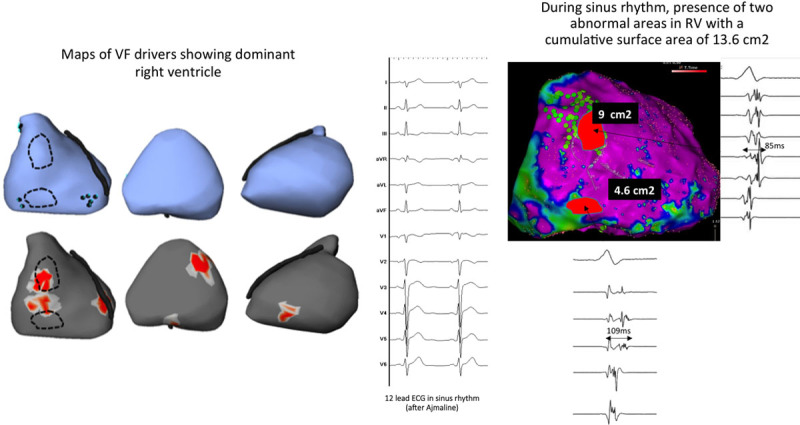
**Example of abnormal signals during sinus rhythm in the right ventricle (RV, patient no. 16). Left**, Maps of ventricular fibrillation (VF) sources, in anterior, inferior, and left lateral views, show focal breakthroughs in the inferior RV (blue points in blue **upper**) and reentry areas in the anterior RV (red areas in **lower**). These sources are largely dominant in the RV. **Middle**, Shows the 12-lead ECG before and after infusion of ajmaline demonstrating the absence of the Brugada pattern (maximal ST elevation is 0.07 mV in V1 or V2). **Right**, Shows the presence of abnormal signals during epicardial mapping in 3 areas in the RV; note also the heterogeneous signal timing indicating localized conduction abnormality. Abnormal signals are recorded within the black dotted contours; which colocated strikingly with those of VF drivers (red areas and blue points). No abnormal electrograms are recorded in the (smaller) left ventricular (LV) driver areas.

The spatial location of structurally abnormal areas was then correlated with the location of VF drivers (mapped in 19 patients). Abnormal areas were found in 13 of these 19 patients; they were found in all 9 patients with drivers dominantly located in 1 ventricle and in 4 of 10 patients with biventricular drivers (*P*=0.013). There was 1 abnormal area in 7 patients, 2 areas in 4, and 3 areas in 2 for a total of 21 areas (Table 2). The spatial relationship of structurally abnormal areas with VF drivers was highly significant (7.4±3.5 mm versus 15.4±3.6 mm; *P*<0.001). Sixteen of these 21 areas (76%) were in close proximity (<1 cm) of a driver region in the right or left ventricle. No colocalization was observed with the septal drivers as only 2 patients presented abnormal areas in the septum.

Figures 3 and 5 show examples of the colocation of structurally abnormal areas and VF drivers. This spatial colocation suggested that the VF mechanism was linked to localized structural alterations in the right or left ventricle, acting as a substrate or anchoring point for reentrant waves.

Subsequent reexamination of the imaging data sets focusing on the abnormal areas failed to identify any structural abnormalities. In addition, all patients having abnormal right ventricular areas had been retested using ajmaline infusion and high electrocardiographic precordial leads which confirmed the absence of Brugada syndrome.

### Ablation

Purkinje triggers were identified in 11 patients. A higher prevalence was observed in patients with a normal ventricular myocardium (Figure III in the Data Supplement) compared with those with abnormal ventricular substrate: 7 of 9 patients (77%) versus 4 of 15 patients (26%); *P*=0.033. Table 2 shows the location of Purkinje triggers and ablation results.

**Figure 6.**
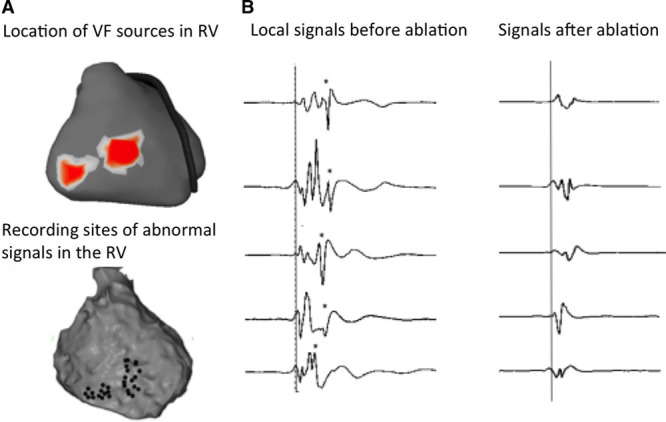
**Effect of ablation on abnormal signals. A**, Location of electrical in the right ventricle (RV, red) during fibrillation in patient no. 19. **B**, Epicardial sites harboring fragmented signals (black points) which delineate the ablation area. Note the contiguity of these sites with the areas of ventricular fibrillation (VF) drivers (red spots). **C**, Baseline signals showing abnormal late fragmented components (stars) and their abolition after radiofrequency ablation (9 min of radiofrequency [RF] delivery).

Catheter ablation targeting the abnormal ventricular substrate was performed in 12 patients with recurrent VF episodes (median, 3.5; IQR, 3–6.25). Three of them had concomitant trigger ablation. The fragmented signals were abolished by 18±5 minutes (median, 19; IQR, 17–21 minutes) of radiofrequency energy applications (Figure 6).

During a median follow-up period of 14 months (IQR, 5.5–19.5 months), no arrhythmic recurrences were recorded on implantable defibrillator monitoring in 15 of 18 patients. Details are provided in Table 2.

## Discussion

This is a first study reporting consecutive patients surviving idiopathic VF who were investigated with a combination of multielectrode body surface recordings during VF and detailed invasive catheter mapping during sinus rhythm. First, we identified the VF drivers and observed that they may be clustered in distinct regions. Then, we found that localized myocardial alterations were present in most patients using established electrophysiological criteria. Finally, there was a colocation of abnormal areas with VF drivers and clinical recurrences of VF were low after driver ablation. Altogether, the findings provide evidence that localized structural alterations underlie a significant subset of previously unexplained SCD.

SCD, a leading cause of death, is mainly related to ischemic heart disease and cardiomyopathies. However, SCD may be unexplained particularly in younger patients, as defined by negative extensive testing excluding structural cardiac disease and primary arrhythmia syndromes. Electrical disorders have been researched using genetic testing, leading to the discovery of many ion-channel mutations, which however concern only a part of affected individuals.

In experimental models, VF is difficult to induce when the hearts are structurally normal. High-resolution mapping showed that VF requires the continuous formation of reentry for its maintenance, of which a critical determinant is the presence of structural heterogeneities. Structural heterogeneities can promote reentry by decreasing conduction velocity and providing a reentrant path or by anchoring reentrant waves. Their role has been shown in multiple conditions, such as physiological (fiber arrangement) or pathological (fibrosis) structure, or by creating experimental heterogeneities.^32–35^ Importantly, a pattern of patchy fibrosis appeared more arrhythmogenic than diffuse fibrosis.^33^ In human cardiomyopathic hearts, reentry has been shown to self-perpetuate for many cycles at the border of myocardial scar.^22,25,26^ The present study suggests a similar physiopathologic link in a subset of idiopathic VF. Activities driving VF were located using body surface recordings, which showed a macroscopic view of epicardial ventricular wave fronts.^20^ Surprisingly, a spatial clustering of activities was observed in some patients, rather than a widespread distribution as expected in structurally normal hearts. Using invasive endo-epicardial mapping in sinus rhythm, abnormal electrograms fulfilling criteria defining structural cardiomyopathy were identified in 62% of patients. They colocated with the VF drivers in the right or left ventricular free walls, but not however with those projecting on the septum. The septal structure includes complex myocardial fiber arrangement, Purkinje tissue, and insertion of papillary muscles and this complexity has been shown to be able to maintain fibrillatory activities without additional pathology.^34,36^

The highest prevalence of structural abnormalities was found in the right ventricle despite normal imaging or pharmacological testing which excluded Brugada syndrome or arrhythmogenic right ventricular cardiomyopathy. However, some of these patients may have a preclinical form of a disease which may manifest later. Endo-epicardial mapping suggested that the pathology involved a part of the ventricular wall (particularly epicardium) rather than being transmural, and only covered a limited surface area; which may explain why they were unperceived by structural imaging. Prior experimental studies have shown that small ventricular lesions (created by thermal or ischemic injury) in the range of 4 cm^2^ are sufficient to promote VF inducibility.^37,38^ The pathology affecting myocardial cells or their connections^39^ was undetermined. The right ventricular epicardium has however specific molecular properties which favor arrhythmogenicity.^40^ In contrast to 62% of patients presenting abnormal myocardial phenotype, no structural abnormality could be detected in 38% of our patients. Importantly, this subset showed a high prevalence of Purkinje triggers, which suggests a dominant Purkinje electrical pathology.

Provided further confirmation in wider populations, our findings suggest that therapy and prevention of unexplained sudden death may be significantly improved by focusing on localized structural alterations. They are in keeping with recent whole-exome genetic studies which showed an increased prevalence of cardiac structure-genes variants, in addition to those affecting ion-channel function.^3,6,12,14^ Patients with idiopathic VF are currently treated with an implantable cardiac defibrillator, justified by the recurrence rate of ventricular arrhythmias, varying from 11% to 45%.^41^ Our study suggests that the ventricular substrate may be a potential target for therapy, particularly when no trigger has been recorded.

This study is subject to several limitations. Our study enrolled a limited number of patients which limit the generalizability of findings. However, their clinical profile—mean age, male dominance, and death during sleep or rest—is strikingly similar to that commonly reported in unexplained sudden death.^10^ Body surface mapping used reconstruction algorithms based on mathematical solutions subject to uncertainties; this limitation was alleviated by analyzing electrogram activation sequence.^22^ The electrophysiological criteria that we used to define structural abnormality are well established and are based on prior largely quoted studies.^26–28,30^ However, these studies have involved a relative paucity of presumed healthy patients, particularly to define normal electrogram criteria in the epicardium, as pericardial mapping in control groups raises an obvious ethical issue. Such patients were thus investigated for idiopathic ventricular premature beats and were older than our cases (47 years^27^). We used multielectrode catheters with 2-mm bipoles which provided high-resolution mapping but also narrower electrogram duration that possibly resulted in an underestimation of abnormal areas.^29^ Finally, the lack of a control group without ablation did not allow to confirm the efficacy of substrate ablation.

In conclusion, this study provides evidence that localized cardiomyopathic areas may underlie a subset of previously unexplained SCD. In other patients, Purkinje triggers seem as a dominant mechanism.

## Sources of Funding

This study was supported by the National Research Agency (ANR-10-IAHU04-LIRYC), the European Research Council (FP7/2007–2013 grant agreement number 322886, SYMPHONY), and the Leducq Foundation (RHYTHM network).

## Disclosures

The authors affiliated to IHU Liryc disclose a Research Collaboration on noninvasive electrocardiographic imaging between Medtronic and the IHU Liryc.

## Supplementary Material

SUPPLEMENTARY MATERIAL
