# Supplementary material for: Localized Structural Alterations Underlying a Subset of Unexplained Sudden Cardiac Death
Source: Circ Arrhythm Electrophysiol. 2018 Jul 12;11(7):e006120. doi: 10.1161/CIRCEP.117.006120 (PMC7661047; doi:10.1161/CIRCEP.117.006120)
Supplement: SUPPLEMENTARY MATERIAL [file hae-11-e006120-s001.pdf]

SUPPLEMENTAL MATERIAL

Patients
Patients were included in the study for primary VF e.g. for resuscitated sudden death due primarily to ventricular fibrillation, excluding those secondary to ventricular tachycardia, ventricular pre-excitation, acute coronary ischemia, and repolarization abnormality. The latter included long QT and short QT syndromes as well as early repolarization syndrome. Early repolarization was defined as an elevation of the terminal portion of the QRS complex of ≥0.2mV in ≥2 contiguous inferior (II, III, and aVF) or lateral (I, aVL, and V4–V6) leads, manifested as QRS notching or slurring.

Idiopathic ventricular fibrillation (IVF) was defined by the absence of other conditions (coronary and myocardial disease) before ICD implantation. Cardiac magnetic resonance imaging, including late gadolinium enhancement, was performed to assess LV and RV volumes and ejection fraction, regional wall motion abnormalities and presence and location of LGE.

In addition to the investigations initially performed at the referral center, all patients had a new Ajmaline test (1mg/kg body weight in 5 min) to exclude Brugada syndrome.Brugada syndrome was defined as an elevation of the ST junction >0.1mV and upward sloping of the ST segment in V1 or V2 leads, including high intercostal lead positioning. All patients had also Isoprenaline test to exclude catecholaminergic polymorphic VT or arrhythmogenic right ventricular cardiomyopathy (Supplem Ref-Denis et al).

Five patients referred for supposed IVF (with documented VF at the index event) were excluded. In two, monomorphic ventricular tachycardia was induced and VF was considered as secondary to VT. One patient had catecholaminergic VT confirmed by Isoprenaline and genetic testing..One patient had left ventricular midwall fibrosis on MRI re-interpretation. One patient had ST elevation and VF during isoprenaline testing, later confirmed to be due to right coronary spasm.

During the same inclusion period, , 25 patients with Brugada syndrome, 14 patients with ischemic heart disease (outside acute MI), and 18 with (dilated, hypertrophic, arrhythmogenic) have been referred.

Genetic analysis
Sequencing was performed on a set of 98 arrhythmia and cardiomyopathy genes using the Haloplex capturing system in 13 patients and fourwas tested for long QT (including SCN5A) genes by standard Sanger sequencing . The following genes were analysed (Supplem ref- Le Scouarnec et al):
ABCC9, ACTC1, ACTN2, AKAP9, ANK2, ANKRD1, ATP2A2, BAG3, CACNA1C, CACNA1D, CACNA2D1, CACNB2, CALR3, CASQ2, CAV3, CSRP3, CTF1, DES, DPP6, DSC2, DSG2, DSP, DTNA, EMD, EYA4, FGF12, FXN, GATA4, GJA1, GJA5, GJC1, GLA, GPD1L, HCN4, HEY2, IRX3, JPH2, JUP, KCNA5, KCNAB2, KCND3, KCNE1, KCNE1L, KCNE2, KCNE3, KCNH2, KCNJ2, KCNJ5, KCNJ8, KCNQ1, LAMP2, LDB3, LMNA, MYBPC3, MYH6, MYH7, MYL2, MYL3, MYLK2, MYOZ2, MYPN, NDUFV2, NEXN, NKX2.5, NOS1AP, NPPA, NUP155, PKP2, PLN, PRKAG2, PSEN1, PSEN2, RANGRF, RBM20, RYR2, SCN10A, SCN1B, SCN2B, SCN3B, SCN4B, SCN5A, SGCD, SLC8A1, SNTA1, SRL, TAZ, TBX20, TCAP, TGFB3, TMEM43, TNNC1, TNNI3, TNNT2, TPM1, TRDN, TRPM4, TTN, VCL

Electrophysiology and Recording Protocol
Antiarrhythmic drugs were discontinued more than 5 half-lives prior to the electrophysiological study in 7 patients. One patient was treated by Amiodarone.

Surface electrocardiographic leads and bipolar intracardiac electrograms, filtered at 30 to 500 Hz, were recorded and stored on a digital amplifier/recorder system (Labsystem , Bard -Boston Billerica,MA). Three catheters were introduced percutaneously through the femoral veins and advanced for endocardial simultaneous recording into the right (RV) and left (LV) ventricle either by retrograde aortic or transseptal approaches: one quadripolar catheter was used for programmed stimulation and two 10 or 20 electrode-catheters (Xtrem, Sorin Medical, or Decanav , Biosense-Webster) to provide electrograms from the right and left ventricles. The RV catheter was placed along the septum or paraseptal anterior wall

with distal tip in the RV apex region. The LV catheter was placed also in the longitudinal LV axis (parallel to the septum).

Epicardial mapping was performed via subxyphosternal access. We used multispline catheters with a 2mm bipolar interelectrode distance for more detailed mapping: either Lasso or Pentaray catheters (Biosense Webster).
Programmed ventricular stimulation was performed using a train of pulses at an initial basic cycle length of 600,then 400 ms, followed by up to 3 extra stimuli until reaching tissue refractoriness. A 25 mA pulse intensity was utilized to try inducing VF during programmed stimulation rather than the more common 2 times threshold intensity. This was done to achieve shorter coupling intervals and facilitate induction by programmed stimulation rather than by burst pacing, which induced early fragmentation of the electrograms precluding quantitative analysis of activation patterns.

In the case of failure of VF induction by programmed stimulation, burst pacing was performed at 250ms pacing cycle length decremented by 10 ms, until 180ms cycle length or 2:1 capture. If stimulation failed to induce sustained VF, the protocol was repeated using pacing from left ventricle. Five patients were not inducible even with burst pacing.

Ventricular fibrillation was considered to be inducible if it lasted for more than 10seconds. ICD therapies were turned off to avoid inappropriate discharge during the VF induction protocol. VF was recorded during external defibrillator charge before shock delivery.

Body-surface potentials
The non-invasive mapping technique used an array of body surface electrodes and CT-based cardiac geometry from a non-invasive mapping system (ECVue™, Medtronic). Briefly, a 252-electrode vest was applied to the patient's torso and connected to the system to record unipolar surface potentials. This was followed by a non-contrast thoracic CT scan to obtain high-resolution three-dimensional images of the individual biventricular geometry and the relative electrode positions via segmentation. The electrode vest could be left up to 8 hours to capture events without altering recording quality; this allowed to capture spontaneous occurrence of VF in 3 patients.

The system reconstructed biventricular surface potentials from unipolar torso potentials using specific reconstruction algorithms (Supplem ref- Rudy et al). All potentials acquired during the initial 5s of VF were exported for further analysis. Maps of VF were generated using specific algorithms combining filtering to eliminate artefacts in signal morphology, and phase mapping. The phases of wave propagation were color-coded. A surrogate of the depolarization and repolarization wavefronts were computed from the isophase values equal respectively to $\pi/2$ and $-\pi/2$. This mapping technique facilitates detection of high-curvature waves, which may create false rotations (22). The reentries were therefore validated by combination with activation mapping e.g. confirmed by sequential activation of raw unipolar local electrograms covering >75% of cycle length. Focal breakthroughs were validated by a QS or small initial R wave pattern for electrogram morphology. Focal breakthroughs were so named without inferring whether the mechanism was truly focal or emergent intramural reentry, a limitation inherent to all clinical techniques, which can only explore some of the ventricular components.

The VF drivers were classified into focal breakthrough, or reentrant (potentially, a rotor). Focal breakthroughs were presented as individual blue points and reentry areas was reported in red areas on the 3D geometries. The cumulative activities in 5sec-duration were quantified and their spatial density displayed in a map in the form of red spots (re-entry) and blue points (focal breakthroughs).

The CT-based individualized biventricular geometry was divided into 3 compartments to classify driver locations (RV, LV, Septum-figure 1B) and RV/LV were further divided in 2 equal parts (RV anterior and RV inferior, LV lateral and LV inferior- figure S1) for a more precise localization of small abnormal electrogram areas. Although the septum could not be directly visualized from the torso, septal activities could be inferred from their projection on the interventricular groove. A compartment harboring more than 50% of total number of activities (e.g. more than the sum of other 2 compartments) was considered

as dominant.

Electrogram fractionation was observed endocardially and epicardially, usually after 5 seconds of VF (in parallel with VFCL shortening). Because this rendered ambiguous the manual electrogram annotation to validate reentry, only the initial 5 seconds were taken for VF analysis.

Frequency –cycle length of ventricular fibrillation
Fibrillation cycle-lengths were measured on body-surface from the mid part of right and left ventricles (excluding the septum) as a mean value for the initial 5 seconds.

Catheter Ablation
Patients were admitted for ablation when they had multiple VF, in keeping with the guidelines. Triggering ectopy, when present, were targeted at their Purkinje or myocardial origin. Structural alterations identified by abnormal electrograms in sinus rhythm were targeted when present. Substrate-based radiofrequency ablation was performed with the target of elimination of abnormal electrograms, as described for ventricular tachycardia. An irrigated-tip quadripolar catheter with a distal 3.5-mm tip and three 1-mm proximal electrodes (Smartouch or Thermocool®, Biosense-Webster) was used for ablation.RF energy was delivered with a power of 30-40 W using irrigation rates of 5 to 60 mL/min (0.9% saline via Cool Flow; Biosense-Webster). RF application had duration varying from 15 to 60 seconds guided by impact on the local electrogram. The temperature was limited to 45°C.

Supplemental Figures

Figure S1 Flow diagram of the study patients

Figure S2 We divided the epicardial surface into six regions to describe more precisely the location of (small) abnormal areas. 1, superior (anterior) right ventricle. 2, inferior right ventricle. 3, inferior left ventricle. 4, superior (lateral) left ventricle. 5, anterior septum. 6, inferior septum.

Figure S3 We analyzed the spatial relationship between structurally abnormal areas (white dots) and VF drivers (red areas) by comparing their mutual distances. The electro-anatomical mapping was registered to the biventricular mesh obtained from the CT scan, which was composed of 1406±17nodes and included the VF driver regions shown here for a selected patient (left panel: RV, middle panel: inferior interventricular groove, right panel: LV). The geodesic distances from all nodes with normal electrograms to the closest driver region border were compared to those from nodes with abnormal electrograms.

Figure S4 Example of normal (not fulfilling the pathology criteria) but fragmented signals during sinus rhythm. Left panel: Maps, in anterior, inferior and left lateral views, show few focal breakthroughs (blue points in upper blue panels) and widespread reentry areas (red areas in lower panels). The pattern of VF activities shows no specific distribution. The middle panel shows a premature ventricular beat on the 12 lead ECG that was later identified from the left posterior Purkinje. The right panel shows the presence of fragmented signals < 70ms in duration in all sites of endocardium and epicardium, defined as normal myocardial tissue.

Figure S1

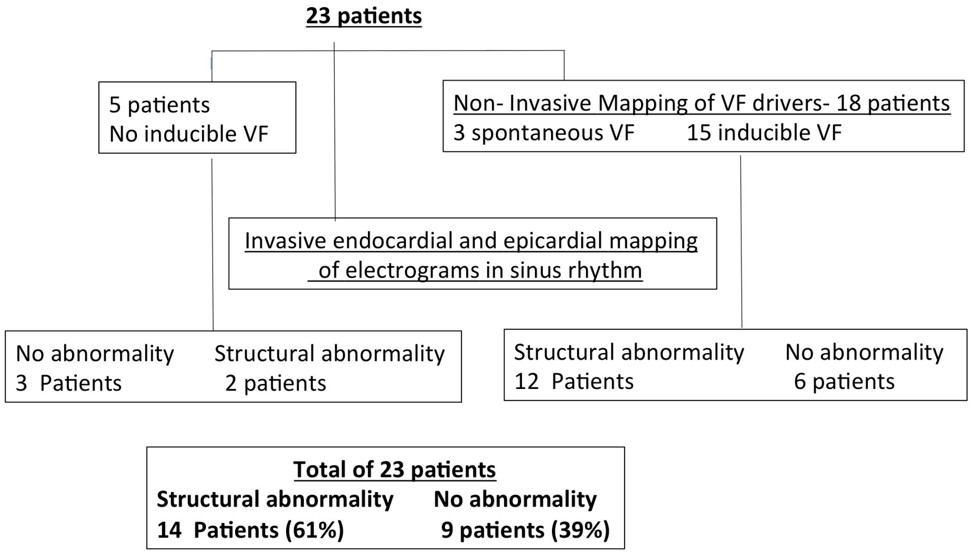
23 patients
5 patients
No inducible VF
Non- Invasive Mapping of VF drivers- 18 patients
3 spontaneous VF
15 inducible VF
Invasive endocardial and epicardial mapping of electrograms in sinus rhythm
No abnormality
3 Patients
Structural abnormality
2 patients
Structural abnormality
12 Patients
No abnormality
6 patients
Total of 23 patients
Structural abnormality
14 Patients (61%)
No abnormality
9 patients (39%)

Figure S2

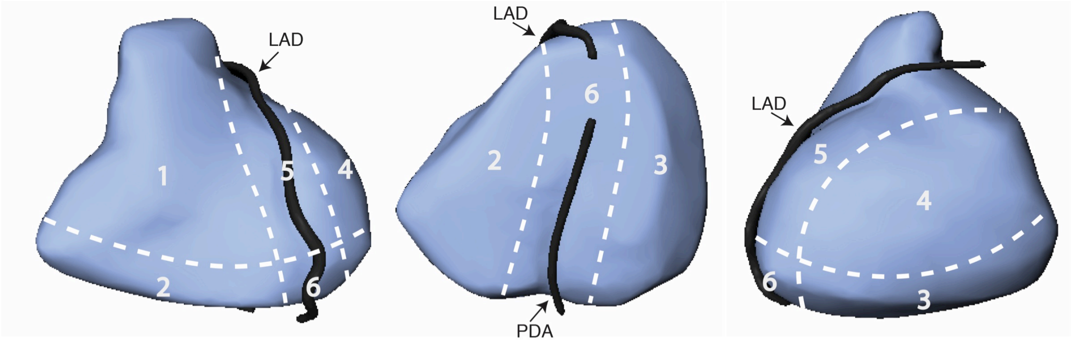

Figure S3

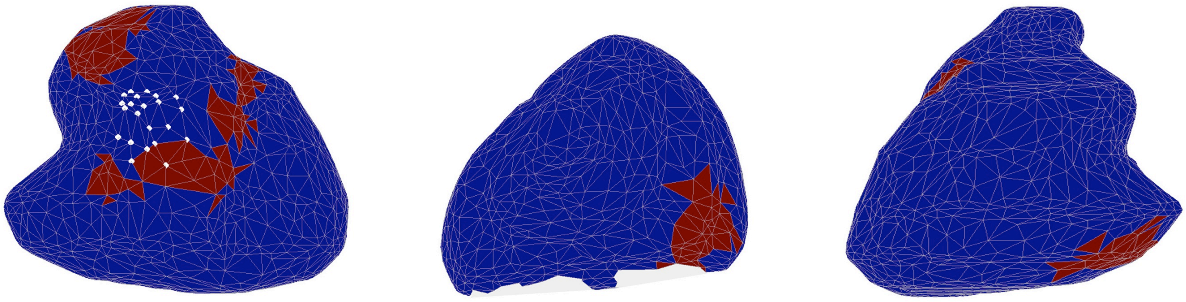

Figure S4

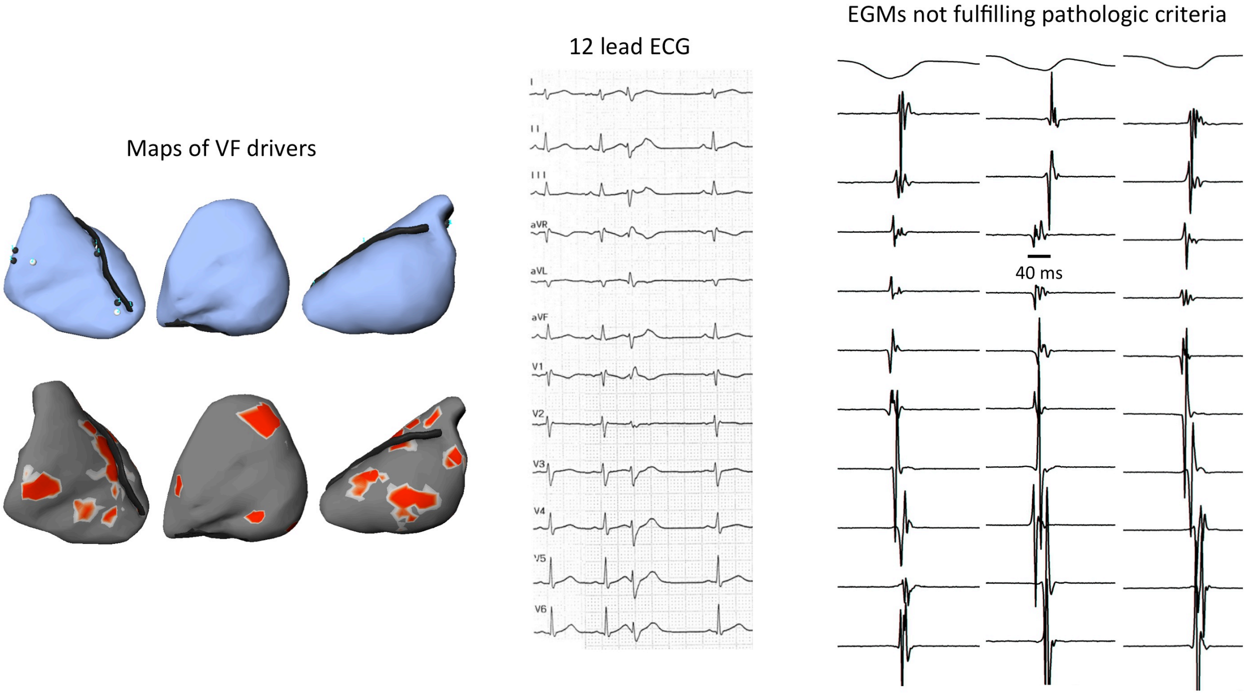
Maps of VF drivers
12 lead ECG
I
II
III
aVR
aVL
aVF
V1
V2
V3
V4
V5
V6
EGMs not fulfilling pathologic criteria
40 ms

Supplemental Movies

Movie S1: Animated wave fronts during the entire VF recordings for Patient #4 showing a right dominant ventricle. Two perspectives are shown (as in Figure 1A) whereby the anterior view (left movie) shows the right ventricle and the left lateral view (right movie) shows the left ventricle. The blue waves (outlined by white lines) in the movies indicate activation waves, which originate dominantly from the right ventricle; while the left ventricle is passively activated (corresponding to figure 1A).

Movie S2: Animated wave fronts during the entire VF recordings for Patient #5 showing a biventricular distribution of sources (no dominant ventricle). Two perspectives are shown (like in Figure 1C) whereby the anterior view (left movie) shows the right ventricle and the left lateral view (right movie) shows the left ventricle. The blue waves (outlined by white lines) in the movies indicate activation waves, which originate from both ventricles (corresponding to Figure 1C).

Supplemental References

Denis A, Sacher F, Derval N et al. Diagnostic value of isoproterenol testing in arrhythmogenic right ventricular cardiomyopathy. Circ Arrhythm Electrophysiol. 2014; 7:590-7

Rudy Y, Messinger-Rapport BJ. The inverse problem in electrocardiography: solutions in terms of epicardial potentials. Critical reviews in biomedical engineering 1988; 16:215-68.

Le Scouarnec S, Karakachoff M, Gourraud J-B et al. Testing the burden of rare variation in arrhythmia-susceptibility genes provides new insights into molecular diagnosis for Brugada syndrome. Hum Mol Genet. 2015; 24:2757–2763
