# Supplementary figures and images for: Localized Structural Alterations Underlying a Subset of Unexplained Sudden Cardiac Death
Source: Circ Arrhythm Electrophysiol. 2018 Jul 12;11(7):e006120. doi: 10.1161/CIRCEP.117.006120 (PMC7661047; doi:10.1161/CIRCEP.117.006120)

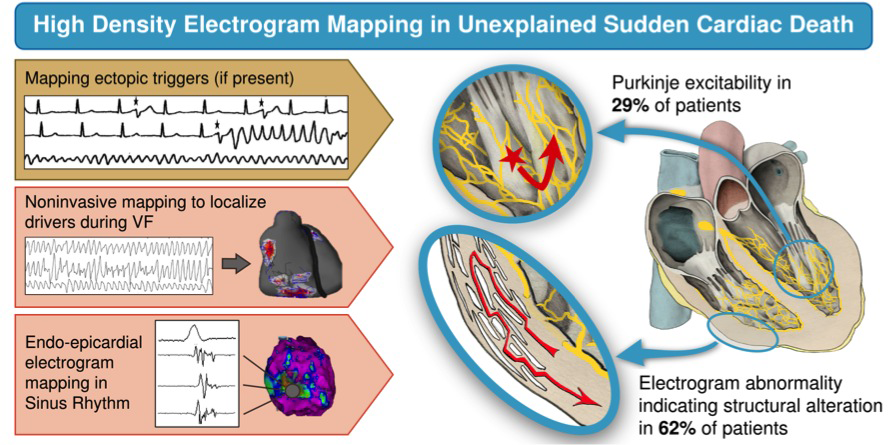

Supplement: SUPPLEMENTARY MATERIAL [file hae-11-e006120-s004.tif]
